# Non-AUG translation initiation in mammals

**DOI:** 10.1186/s13059-022-02674-2

**Published:** 2022-05-09

**Authors:** Dmitry E. Andreev, Gary Loughran, Alla D. Fedorova, Maria S. Mikhaylova, Ivan N. Shatsky, Pavel V. Baranov

**Affiliations:** 1 Shemyakin-Ovchinnikov Institute of Bioorganic Chemistry, RAS, Moscow, 117997 Russia; 2 Belozersky Institute of Physico-Chemical Biology, Lomonosov Moscow State University, Moscow, 119992 Russia; 3 School of Biochemistry and Cell Biology, University College Cork, Cork, T12 XF62 Ireland

## Abstract

Recent proteogenomic studies revealed extensive translation outside of annotated protein coding regions, such as non-coding RNAs and untranslated regions of mRNAs. This non-canonical translation is largely due to start codon plurality within the same RNA. This plurality is often due to the failure of some scanning ribosomes to recognize potential start codons leading to initiation downstream—a process termed leaky scanning. Codons other than AUG (non-AUG) are particularly leaky due to their inefficiency. Here we discuss our current understanding of non-AUG initiation. We argue for a near-ubiquitous role of non-AUG initiation in shaping the dynamic composition of mammalian proteomes.

**Supplementary Information:**

The online version contains supplementary material available at 10.1186/s13059-022-02674-2.

## Introduction

The initiation of translation of most eukaryotic mRNAs occurs via the so-called scanning mechanism which was championed by Marylin Kozak [1, 2]. The start codon, usually AUG, is recognized by the preinitiation complex (PIC) consisting of a 40S ribosomal subunit loaded with eukaryotic initiation factors (eIFs) and Met-tRNA_i_. The PIC first enters at the 5′ end of mRNA via recognition of m^7^G cap and then scans the 5′ “untranslated” region (leader) in the 5′-to-3′ direction. Recognition of a suitable start codon is followed by dissociation of initiation factors and 60S subunit joining (reviewed in [3–5]).

At a mechanistic level, start codon recognition depends on codon-anticodon interaction between AUG and Met-tRNA_i_. During the elongation phase, all codons are decoded in the A-site; however, the start codon is recognized in the ribosome P-site. Base pairing in the P-site is not monitored with the same strictness as in the A-site where the Watson-Crick geometry of the base pairs involving the first two subcodon positions is strictly enforced by the ribosome [6, 7].

Presumably, the reduced control of the base-pairing geometry in the P-site enables Met-tRNA_i_ to recognize codons other than AUG as starts, albeit less efficiently. In addition to the identity of the potential start codon, the likelihood of translation initiation is influenced by the surrounding nucleotide context which has been extensively investigated. Kozak reported that the 6 nt preceding the initiation codon and the nucleotide immediately downstream influence translation initiation efficiency, with GCCRCC**AUG**G (R = A or G) being the consensus sequence in vertebrate mRNAs [8–10]. Specifically, positions −3 and +4 (where +1 refers to A in AUG) were found to be the most important for efficient AUG recognition, with A/G in −3 and G in +4 being optimal. All possible context efficiencies for AUG initiation from −6 to +5 have been recently characterized quantitatively and this analysis finds the strongest context to be RYMRMV**AUG**GC (Y = C or U; M = A or C; V = G, C or A) [11].

Not all mRNAs possess optimal context AUGs at the start of their coding sequence (CDS). Noderer et al. [11] applied a quantitative estimation of all protein coding AUG initiation efficiencies across the human transcriptome and found that although the distribution is shifted toward more efficient translation initiation starts (TISs), thousands of mRNAs possess non-optimal and “weak” context starts. Some of the weakest context initiation codons are highly conserved [12–14]. The difference in initiation efficiencies between optimal and weak naturally occurring AUG contexts is estimated to be up to 12-fold [11]. Suboptimal AUG TISs facilitate leaky scanning and it has been shown that in such cases the next downstream start tends to be located in the same reading frame, thus allowing translation of additional “truncated” proteoforms [15].

More than half of all human mRNAs have at least one AUG codon upstream (uAUG) of their annotated TIS (we searched current transcript annotations and found that 58% in the current versions of RefSeq (annotation release 109.20210226) and 56% in Gencode (version 37)). Their potential use as TISs could result in translation of so-called upstream Open Reading Frames (uORFs). uORF translation usually results in the synthesis of short polypeptides, some of which have been shown to be functional, e.g., in *ASNSD1* [16], *MIEF1* [17–20], *MKKS* [21], and *SLC35A4* [17]. However, it is believed that most translated uORFs only have a mild inhibitory effect on downstream translation [22]. The average inhibitory effect of uORFs is mild either because most uORF starts are leaky (non-AUG or AUG in a weak context), or also because ribosomes terminating after translation of short ORFs are often capable of reinitiating [23].

Nonetheless, translation initiation on most mRNAs likely occurs at more than one AUG. Furthermore, modified ribosome profiling techniques that enrich ribosome footprints at TISs revealed that non-AUG TISs are even more abundant than AUG TISs [24–26], suggesting that TIS plurality is pervasive in mammalian mRNAs. Furthermore, the stringency of start codon selection is plastic and can be regulated by the cellular levels of specific factors [12–14, 27] leading to altered ratios of AUG vs non-AUG initiation which subsequently varies across different (patho)physiological conditions. Despite this growing appreciation of the role of non-AUG initiation in shaping mammalian proteomes, its exploration is still in its infancy. In this review, we provide a glimpse into recent findings and argue for the need for increased awareness of this phenomenon in genome annotations and gene expression studies.

While non-AUG initiation is known to take place in prokaryotes, as well as in lower eukaryotes such as yeast and in plants, this is outside the scope of this review and is discussed elsewhere [28–30]. We mention relevant studies in other eukaryotes only when they provide important mechanistic insights in a historical context.

### Large proteins whose synthesis initiates at non-AUG start codons

**Fig. 1:**
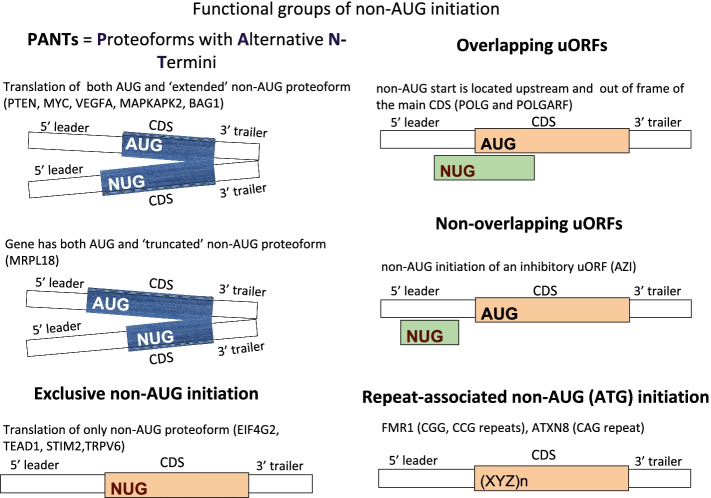
Utilization of non-AUG initiation. Variation of non-AUG initiation with known examples. Non-AUG codons are denoted with NUG (they may differ from AUG in the second and third position)

Depending on its location within a particular mRNA, alternative TISs yield either translation of an alternative ORF or, if it is located in the same reading frame as the main AUG-initiated ORF, it can yield N-terminally extended or truncated proteoforms (Proteoforms with Alternative N Termini, PANTs) (Fig. 1).

One example of non-AUG initiation on a mammalian mRNA was demonstrated for the oncogenic transcription factor *MYC* (a.k.a. c-Myc). Hann et al. [31] detected two c-Myc proteoforms that originate from a single mRNA with a single AUG-initiated ORF. It was subsequently shown that N-terminally extended Myc (p67, myc1) starts from a CUG codon located 15 triplets upstream of the main ORF AUG. The N-terminally extended proteoform of c-Myc differentially regulates transcription through a noncanonical DNA-binding site and thus is functionally distinct from the AUG-initiated proteoform, the latter appears to be more favorable for a phenotype supportive of cancer progression than the CUG-initiated proteoform [32, 33]. Intriguingly, among the few known instances of non-AUG initiated proteoforms, several are expressed from genes implicated in cancer. These include *VEGFA* encoding vascular endothelial growth factors whose extended proteoform is secreted [34], *BAG1* encoding Bcl-2-associated athanogene-1 [35–40], and *FGF2* whose CUG-initiated proteoforms are targeted to the nucleus [41–43].

Ivanov et al. [44] reasoned that the functionality of non-AUG extended proteoforms should constrain their evolution. Searches for sequences upstream of annotated AUG TISs that are evolutionary conserved identified 42 novel non-AUG initiated proteoforms including a CUG-initiated proteoform of the *PTEN* gene whose product is extended by 173 residues. PTEN is a tumor suppressor and an antagonist of the phosphoinositide-3 kinase (PI3K) pathway. It was later reported that the extended proteoform is secreted from cells and can enter other cells to alter PI3K signaling [45]. A subsequent study that focused on identifying the exact TIS revealed several non-AUG TISs in addition to CUG with an AUU being the most efficient [46]. This study also cast doubt on the secretory potential of any N-terminally extended PTEN proteoforms. While such phylogenetic approaches can successfully identify functionally important extensions, several known instances of reported non-AUG TISs are poorly conserved. However, poor conservation does not preclude their phenotypic relevance. For example, contrary to expectations, one of the most extensively studied examples of non-AUG initiation, the above mentioned CUG codon in *MYC*, is absent in several mammals (e.g., substituted to CUA codon in cow, sheep, and some other ungulates).

In the above examples, non-AUG TISs are located upstream of the main AUG start. Detectable non-AUG TISs are more frequent upstream of AUG TISs as expected according to the scanning model of translation initiation as fewer PICs would be available for non-AUG initiation downstream of efficient AUG initiators [47]. Yet, despite this, N-terminally truncated non-AUG initiated proteoforms are known (Fig. 1). A remarkable example was reported for *MRPL18* encoding mitochondrial ribosomal protein L18 [48]. Here initiation at a CUG codon occurs downstream of the main AUG codon under heat shock stress conditions. The truncated ribosomal L18 protein lacks a mitochondrial localization sequence and is instead incorporated into cytoplasmic ribosomes. These “hybrid” MRPL18 containing ribosomes have been proposed to be involved in the translation of stress induced mRNAs.

**Fig. 2:**
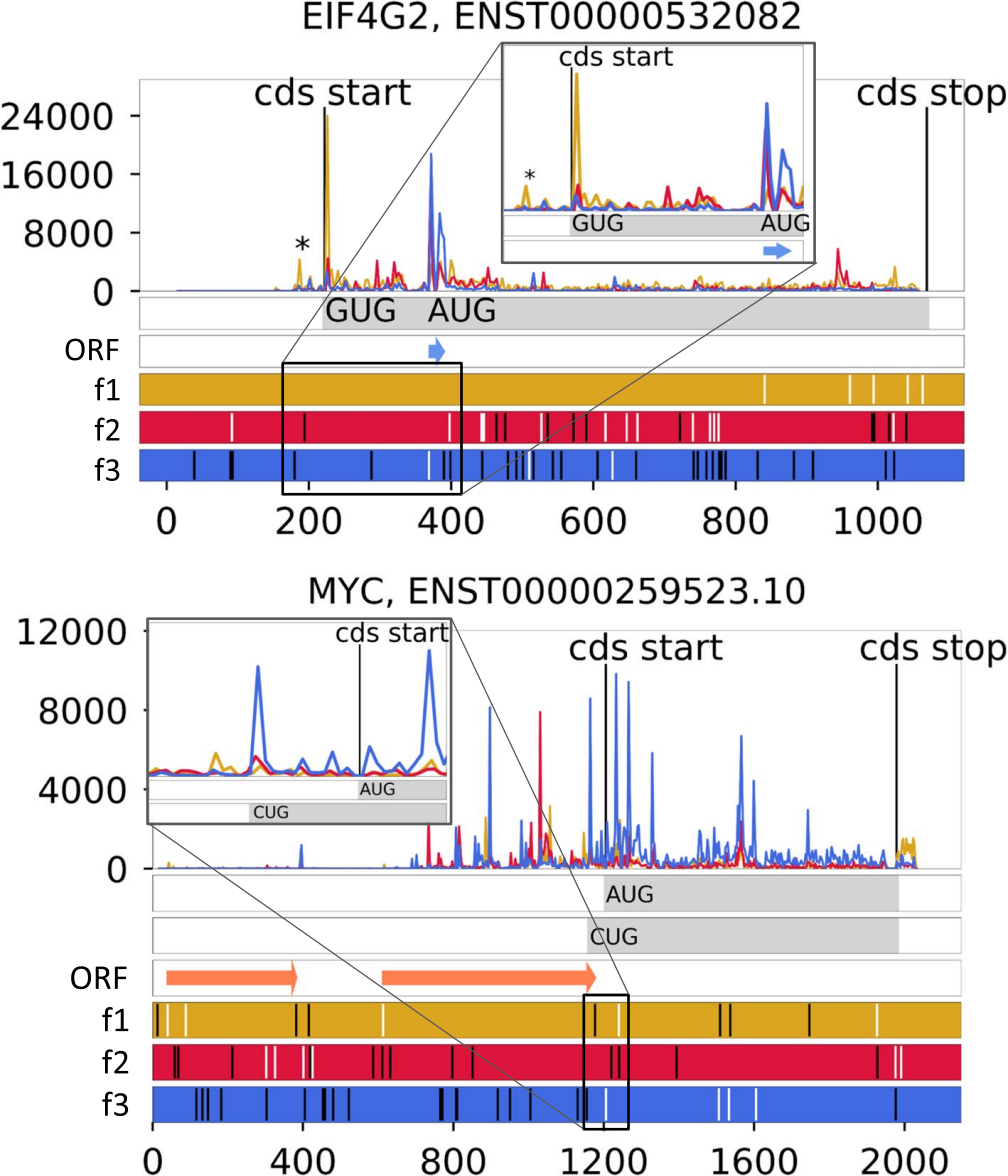
Ribo-seq profiles of *MYC* (example of PANTS: AUG and extended CUG proteoforms) and *EIF4G2* (example of exclusive non-AUG initiation). Reading frames are colored in red, green, and blue; AUGs (white bars) and stop codons (black bars) are shown within the bottom frame color bars. The asterisk (*) in *EIF4G2* represents upstream non-AUG initiation at AUU codon; a downstream out-of-frame short AUG ORF is depicted by a blue arrow; the main ORF (CDS) is shown as a gray bar. In the *MYC* profile, AUG and CUG proteoforms (main ORFs) are shown as gray bars, and AUG uORFs are shown as red arrows

Interestingly, there are some mRNAs where the predominant initiation codon is non-AUG (Fig. 1). The most well-known example is in the *EIF4G2* [49] gene which codes for translation initiation factor eIF4G2 a.k.a. DAP5. Translation of *EIF4G2* mRNA is thought to occur exclusively at a GUG codon, although examination of publicly available ribosome profiling data in Trips-Viz [50] suggests the existence of an additional minor non-AUG TIS shortly upstream (Fig. 2).

Non-AUG initiation is a simple mechanism enabling multiple TISs on the same mRNA, but it does not lead to similar proteoforms with heterogeneous N-termini when it occurs in a reading frame different from the CDS (Fig. 1). An example of a protein encoded by a non-AUG initiated ORF that overlaps the main CDS was recently discovered in *POLG* mRNA [51, 52]. *POLG* encodes the catalytic subunit of mitochondrial DNA polymerase. Initiation at a CUG codon located upstream of the *POLG* CDS yields a long protein (260 amino acids in humans) termed POLGARF. While the functional role of POLGARF is still not clear, there is evidence that POLGARF may participate in extracellular signaling. Ectopically expressed POLGARF can be cleaved and secreted upon serum stimulation [52]. The likely importance of POLGARF is supported by phylogenetic conservation of its overlapping reading frame in placental mammals [51, 52]. Since the *POLGARF* coding ORF overlaps the *POLG* coding ORF by 725 nucleotides, there are implications for the interpretation of *POLG* synonymous mutations.

### Non-AUG initiation of short ORFs (sORFs) translation

While it is likely that more novel proteins and proteoforms of known proteins synthesized as a result of non-AUG initiation are yet to be discovered, it is apparent that the vast majority of non-AUG initiation leads to the translation of sORFs. In fact, ribosome profiling of ribosomes enriched at start codons suggests that translation of most sORFs initiates at non-AUG codons [24, 25] and this was confirmed with proteomic studies [53–55]. Because of leaky scanning, productive non-AUG codons are far more likely to occur upstream of AUG codons [47]. Subsequently most non-AUG sORFs are expected to be located at the beginning of RNA transcripts, though translation of non-AUG initiated ORFs downstream of the CDS TIS have also been reported [56]. What are the functional and phenotypic consequences of translation of these sORFs? It is certainly possible, and even likely, that this abundant but inefficient translation is driven by the high evolutionary cost of optimizing the translational apparatus for accurate translation initiation. However, even if most non-AUG initiation is non-adaptive [57], it is clear that translation of at least some non-AUG sORFs is beneficial as evident from deep evolutionary conservation of some of them. An example is the high frequency of conserved non-AUG initiated uORFs in genes involved in the regulation of polyamine levels [58]. The regulatory roles of many uORFs are well established, with many enabling gene-specific regulation in response to particular conditions, e.g., polyamine [59] or magnesium [60] concentrations as well as during stress [17, 61]. Non-AUG initiated uORFs provide yet another regulatory layer to uORFs because of the apparently dynamic and regulatable stringency of translation initiation as we will see further below. During Oxygen and Glucose Deprivation (OGD), many non-AUG uORFs whose translation was reciprocal to that of its corresponding CDS were found. This coincided with altered translation of mRNAs involved in the regulation of translation initiation stringency [62] supporting their autoregulation discussed in more detail further below.

Even if the products of non-AUG sORFs or their translation have no specific functions, their translation is not inconsequential for the organism’s phenotype. In addition to imposing an energetic burden, since protein synthesis is the most energetically demanding cellular process [63], the products of sORF translation could become a source for antigen presentation and thus contribute to the immune response [64, 65]. Of note, in addition to being recognized by standard Met-tRNA_i_, a Leu-tRNA was identified that can also initiate (inefficiently) at CUG codons [66]. Given the low level of evolutionary selection acting on most non-AUG starts, the repertoire of translated sORFs may vary among individuals that may contribute to personalized immunopeptidomes. Apart from its likely role in shaping the immunopeptidome, promiscuous translation of non-AUG initiation has been implicated in cancer development linking it to cell survival and proliferation [67, 68]. The pathogenicity of non-AUG initiation at sORFs has received particular attention in relation to neurodegenerative diseases associated with repeat expansions, termed RAN (repeat-associated non-ATG) translation. Long repetitive sequences have been shown to stimulate translation initiation upstream; more details on the molecular processes and associated disease etiology can be found in several reviews dedicated to this topic [69–71]. However, it is important to note that during RAN translation, recognition of non-AUG codons seems to occur via the standard cap-dependent mechanism and involves canonical initiation factors and regulators [72–74].

### Nucleotide contexts that influence non-AUG initiation

Perhaps the most important feature, other than mRNA position, that affects non-AUG initiation efficiency is the codon identity. Various approaches have been implemented to investigate initiation efficiencies of near cognate codons including reporter constructs [75] or ribosome profiling of cells treated with antibiotics that enrich for ribosomes at initiation sites [24, 25]. All of these studies reveal that the most efficient non-AUG codon is CUG, which is typically at least 2–3 times more efficient than any other non-AUG codon. It is not clear whether the CUG codon is the most efficient simply due to the minimal topological constrains of its codon-anticodon duplex [76] or for different reasons—we expect that this question will be addressed soon with structural studies of near cognate translation initiation complexes.

As AUG start codon recognition critically depends on its nucleotide context, it is not surprising that context has an even higher influence on non-AUG initiation, as additional forces that stabilize the preinitiation complex facilitate recognition of an inefficient start codon. This implies that non-AUG initiation could be sensitive to nucleotide positions which have little significant influence on initiation at AUG codons. Indeed, early studies based on reporter constructs indicated that nucleotides at +5 and +6 are important for the efficiency of non-AUG initiation (both studies concluding that the optimal nucleotides at positions +5 and +6 are A and U, respectively) [77, 78].

The development of high throughput approaches for massively parallel variant analysis of initiation efficiency, FACS-seq, allowed estimation of all individual context variants in cells. In these studies, libraries of GFP reporters in which several positions around the start codon were randomized were transfected into cells. The cells were sorted and binned by relative expression levels of the reporters before amplification and sequencing. This approach was first implemented for AUG contexts [11], and later extended to non-AUG codons [75]. FACS-seq studies provided new important insights into the determinants of near cognate start codon efficiencies. It directly demonstrated that the optimal context for initiation at non-AUG and AUG codons is not exactly the same. Certain nucleotide contexts enable non-AUG initiation with efficiencies comparable to AUG initiation. These findings challenged the hitherto prevailing view that the efficiency of non-AUG initiation is generally no more than 5–10% of that of an AUG. Another non-trivial observation was the variation of optimal contexts among different non-AUG codons. Certain contexts allow generally less efficient non-AUG codons to outperform the most efficient non-AUG codons (e.g., AUU vs CUG).

Although the above study was limited to the context within −4 to +4 positions and cannot be used to verify the claim about +5 and +6 positions, certain general conclusions can be made regarding the context of non-AUG initiation. Firstly, non-AUG initiation is critically dependent on the +4 nucleotide being guanine. While +4G is also optimal for AUG initiation, adenine in this position is usually tolerated without strong decreases in translation efficiency. In contrast, a +4G/A substitution has a dramatic effect on non-AUG initiation which can decrease by up to 10 times for CUG. Secondly, the −4 position also has a strong influence on near cognate initiation. Nucleotide changes at the −4 position caused the efficiency of non-AUG TISs to vary more than 2-folds, but the efficiency of AUG TISs varies only by ∼10%. In particular, C (or also A, depending on the assay) in the −4 position caused a ∼70% increase in the expression from all non-AUG codons compared to G in the same position. Altogether, with the early findings highlighting the influence of the +5 and +6 positions (which were not addressed in the FACS seq study), it is possible to predict that the most efficient non-AUG initiator (codon and context) as (C/A)(A/G)(C/A)(A/C/G)**CUG**GAU. Analysis of phylogenetic conservation of nucleotide contexts for *EIF4G2*, *R3CCH1*, and *POLGARF* non-AUG contexts (which were shown to achieve 20–40% of initiation relative to AUG) supports the importance of −4, −3, and +4 positions as C, A, and G respectively [52].

Notably, many known non-AUG starts implicated in the synthesis of functional proteoforms do not bear optimal near cognate contexts and hence can be considered as suboptimal non-AUG initiators. For instance, the human *MYC* context (**G**ACG**CUG**G) and *BAG1* context (**G**GGC**CUG**G) do not have −4C, and the *VEGFA* context (CGCG**CUGA**) lacks +4G. On the other hand, the non-optimality of non-AUG initiation is expected from the simple fact that in many cases initiation codons other than CUG are utilized.

### Factors and intracellular conditions involved in global regulation of non-AUG initiation

**Fig. 3:**
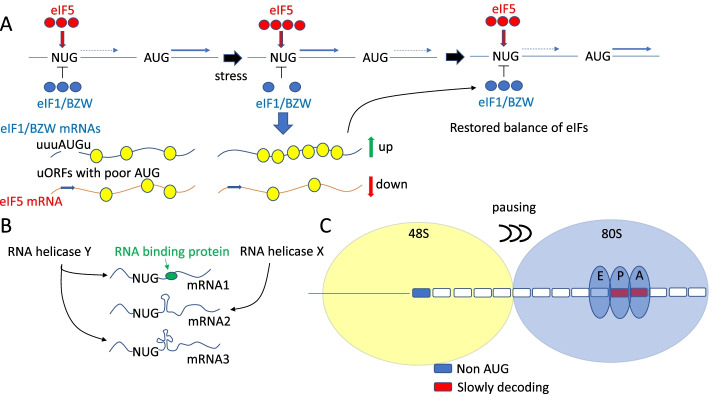
Global and mRNA-specific regulation of non-AUG translation. **A** Global regulation of non-AUG initiation by eIF1/BZW/eIF5 “stringency” factors and the regulatory circuit that ensures their levels are tightly controlled. **B** Downstream secondary structures and/or RNA binding protein sites render mRNA translation sensitive to specific RNA helicase action. **C** Stimulatory effect on non-AUG initiation by a preceding slowly elongating or paused 80S ribosome

In this and the following two sections, we will discuss 3 different ways of non-AUG initiation regulation illustrated in Fig. 3. The first example describes transcriptome-wide regulation by the modulation of the activity/availability of certain initiation factors involved in the stringency of start codon selection (Fig. 3A). The second example outlines changes in the composition of scanning complexes that affect their ability to unwind downstream RNA secondary structures known to stimulate initiation (Fig. 3B). This may selectively affect some initiation sites more than others depending on local RNA structures. The last example requires paused 80S ribosomes positioned downstream (Fig. 3C); again, such regulation is transcript specific.

The idea that some components of the translation apparatus can modulate start codon recognition arose from yeast genetic experiments when the Donahue group performed a genetic screening for sui/SUI alleles (suppressors of initiator codon mutants), taking advantage of these mutants’ ability to confer translation of a mutant his4 allele despite the absence of an AUG start codon. These sui/SUI genes encode initiation factors eIF1, all three subunits of eIF2, and eIF5 [79–81].

eIF1 is a critical factor involved in the stringency of start codon selection. Initial studies from Pestova’s group demonstrated that in a mammalian reconstituted translation initiation system, omission of eIF1 prevents formation of the 48S complex on AUG codons and instead leads to an accumulation of 48S complexes upstream of the AUG [82]. Omission of eIF1 from this in vitro translation system leads to 48S complex formation at UUG and GUG codons located closer to the 5′end of β-globin mRNA than the AUG initiator [83]. The rate of dissociation of eIF1 from 48S seems to be a crucial determinant of start codon recognition [84]. Subsequently, it was shown that accurate translation initiation entails control of binding and release of eIF1 by other factors such as eIF3 [85, 86] and eIF5 [87, 88].

eIF5 also plays an important role in scanning and start codon selection [89, 90]. During the scanning process, eIF5 stimulates hydrolysis of GTP in the ternary complex (TC). However, the release of the phosphate (P_i_) from eIF2-GDP-P_i_ is prevented until the start codon is recognized. Upon start codon recognition, the N-terminal domain of eIF5 occupies the eIF1 binding site on the ribosome and prevents eIF1 rebinding thus stabilizing the codon-anticodon interaction and promoting initiation [91].

The *BZW2* and *BZW1* genes encode translation regulatory proteins termed eIF5 mimic proteins 5MP1 and 5MP2 respectively. Both proteins are homologous to eIF5 at its C-terminal domain but lack its N terminal GTPase activating GAP domain. Like eIF5, 5MP1/2 can bind eIF2, but due to the absence of GAP activity they act as dominant negative translation repressors [13, 92].

Both eIF1, eIF5, and 5MP1 and 2, when overexpressed in mammalian cells, affect initiation at suboptimal codons, including non-AUG codons, at least with reporter constructs. Specifically, eIF1, 5MP1, and 5MP2 decrease initiation on suboptimal codons, while eIF5 stimulates it [14, 27]. These factors are likely very prominent in the regulation of non-AUG initiation at the genome wide level. As mentioned earlier, their own synthesis is autoregulated *via* a network of evolutionary conserved negative feedback loops. The initiation contexts of the *BZW1/2* and *EIF1* AUG codons are very weak, and high levels of 5MP1/2 or eIF1 (increases stringency) suppressing translation of their own mRNAs [12, 13]. The 5′ leader of the eIF5 mRNA contains several inhibitory uORFs with evolutionary conserved poor initiation contexts [27]. Consequently, high levels of eIF5 (reduces stringency) also suppresses translation of its own mRNA by increasing translation of its inhibitory uORFs. This regulatory network (Fig. 3A) is interconnected, and these factors can also regulate each other’s translation, e.g., increased eIF1 levels enhance eIF5 translation and vice versa. This conserved genetically encoded circuit likely serves to ensure stringent start codon selection appropriate for specific conditions.

In agreement with this, depletion of eIF1 in cells showed a somewhat modest effect on global translation [93]. Indeed, upon eIF1 deprivation, only 245 out of 10,000 actively translated transcripts showed changes in translation. As expected, under these conditions eIF1 depletion resulted in a concomitant decrease in eIF5 translation, thus restoring the balance of “stringency” factors and limiting the global effect on translation from suboptimal start site initiations [93]. As mentioned earlier, the cell response to metabolic stress such as OGD illustrates this regulatory system in action [62]. OGD leads to an increase of translation in 5′ leaders which includes increased initiation at some non-AUG codons, which may indicate a relaxed stringency of translation initiation. Interestingly, after 60 min of OGD, *EIF5* is translationally repressed and this is accompanied by a marked increase in *EIF1* mRNA translation. This change is expected to alter the balance between eIF1 and eIF5 in order to make initiation more stringent in response to the stress. Notably, eIF1B, the paralog of eIF1 which shares 92% sequence identity, may also be implicated in start codon selection and the regulation of start codon selection stringency [93].

### Helicase apparatus and its interplay with non-AUG initiation

In her pioneering work, Kozak proposed that RNA secondary structures located downstream of sub-optimal starts can enhance initiation [94]. The stimulatory effect of stem-loop structures is position dependent with an optimal location 14–16 nt downstream of a start codon which roughly corresponds to the region occupied by the ribosome based on RNase protection [95–97]. The most likely explanation is that a scanning ribosome complex pauses while unwinding the secondary structure, which positions the ribosome P-site close to the start codon thus increasing the dwell time at this codon and subsequently increasing the probability of start codon selection. In line with this observation, it has been recently shown that translation initiation of a CUG-initiated protein POLGARF is stimulated 3-fold by a 3′ RNA stem loop. In combination with an efficient nucleotide context, it makes this CUG codon one of the most efficient non-AUG start sites with efficiencies up to 60–70% of AUG [52].

Whereas, in general, downstream structures can enhance non-AUG initiation, they vary and may respond differently to the compositions of scanning complexes. While it seems that the core helicase, eIF4A, is critical for scanning [98, 99], additional helicases and accessory factors are involved in the unwinding of specific structures upon scanning [100]. The unwinding of such “difficult” structures may increase dwell time of scanning ribosomes in the absence of corresponding factors and hence increase initiation at upstream non-AUG codons. This raises the intriguing idea that specific secondary structures downstream of suboptimal start codons can render non-AUG initiation sensitive to a specific RNA helicase activity. For instance, Murat and colleagues [101] investigated the role of two accessory RNA helicases, DHX36 and DHX9, in human cells using ribosome profiling. They demonstrated that depletion of the rG4-quadruplex unwinding helicases DHX36 and DHX9 promotes translation of rG4-associated uORFs while reducing the translation of their corresponding CDS. Mechanistically, these helicases assist in the unwinding of G quadruplexes (and perhaps other structures) which otherwise serve as roadblocks for scanning ribosomes, and upon helicase depletion such structures can promote initiation at suboptimal codons. A striking example of such a mechanism was demonstrated for *DDX23* mRNA translation, which is dependent on DHX9 helicase. A specific RNA motif located in the *DDX23* 5′ leader, which binds DHX9, is located upstream of an AUG-initiated short uORF. siRNA-mediated depletion of DHX9 cause increased translation of this uORF which is accompanied by a decrease in its CDS translation [101].

It is reasonable to propose that similar mechanisms can also operate for non-AUG codons. Indeed, direct evidence of such a mechanism has come from experiments with yeast. Guenther and colleagues [102] investigated the role of DEAD-box RNA helicase Ded1p (DDX3 is its mammalian ortholog) in translation. Whereas Ded1p regulates global translation, some mRNAs are more sensitive to Ded1p depletion. It was further demonstrated that Ded1p depletion leads to increases in initiation at non-AUG codons located immediately upstream of RNA secondary structures.

In principle, similar initiation-inducing roadblocks can be created by RNA binding proteins. One prominent example is the regulation of *msl-2* mRNA translation by Sex lethal (SXL) protein, which is critical for dosage compensation in Drosophila. Medenbach and colleagues [103] demonstrated that SXL binding downstream of a short uORF imposes a strong negative effect on main ORF translation, by increasing initiation at the uORF and augmenting its impediment to downstream translation. It is reasonable to expect that this mechanism will also operate for non-AUG codons and that specific RNA helicases may be required to displace a particular RNA binding protein during scanning. Thus, nucleotide sequence motifs located immediately downstream of non-AUG codons may confer specificity of mRNA translation to various accessory helicases and/or RNA binding proteins, and this allows protein-specific translation control of non-AUG initiation for selected mRNAs (Fig. 2B).

### Interplay between elongation and initiation rates can be important for non-AUG initiation

Like RNA structures and RNA binding proteins, a paused elongating 80S ribosome could also provide a stimulatory effect on initiation upstream. Such a possibility was initially proposed to occur during translation of barley yellow dwarf virus RNA [104] and is likely responsible for an observed increase in non-AUG initiation transcriptome-wide in response to mild cycloheximide treatment, which slows down, but does not completely inhibit elongation [105]. This was shown not only using reporters, but also on endogenous mRNAs, e.g., initiation at GUG in *EIF4G2* mRNA. Whereas this effect was observed under artificial conditions of antibiotic treatment, it is possible that similar effects can be realized under physiological conditions when the elongation rate is significantly decreased. Global translation elongation rates are controlled *via* eEF2K-mediated phosphorylation of eEF2. eEF2K is a calmodulin-dependent kinase which phosphorylates eEF2 on T56 and prevents its binding to the ribosome [106–110]. Many signaling pathways control eEF2 activity indirectly by regulating the activity of eEF2K. For example, SAPK4/p38delta [111], p90(RSK1), and p70 S6 kinase [112] phosphorylate eEF2K and inhibit its activity, while AMP-activated protein kinase (AMPK) activates eEF2K [113, 114]. It is therefore reasonable to propose that eEF2K-mediated eEF2 phosphorylation, which occurs not only upon various stress conditions but also during mitosis [115], can globally enhance non-AUG initiation in a way that is reminiscent of low dose cycloheximide treatment. However, as stress conditions may also result in decreased initiation, this can counteract ribosome queue formation caused by slow elongation.

This mechanism implies that slowly decoded sequences downstream of a suboptimal codon could selectively enhance non-AUG initiation. Indeed, such an example was reported for the *AZIN1* mRNA which codes for antizyme inhibitor, a key regulator of polyamine biosynthesis. Elevated polyamine levels repress translation of the *AZIN1* main ORF, and this is dependent on the presence of an AUU-initiated inhibitory uORF [58]. A subsequent study revealed the mechanism of uORF mediated translation control—a PPW motif encoded at the C-terminus of the uORF promotes ribosome stalling when polyamine levels are high, and this stalling enhances initiation at the uORF AUU codon ~30 codons upstream [116], thus confirming Dinesh-Kumar’s [104] hypothesis and providing a possible mechanistic explanation for the later observation of an increase in global non-AUG initiation in response to elongation inhibitors [105].

Site-specific ribosomal pauses can control the use of non-optimal start codons at a genome-wide level. eIF5A is a highly conserved translation factor which functions during both translation elongation and termination. During translation elongation, it facilitates peptide bond formation between amino acids where the peptidyl transfer reaction is thought to be inefficient [117–119]. Manjunath and colleagues [120] showed that depletion of eIF5A enhances upstream translation within some 5′ leaders across yeast and human transcriptomes. Among those mRNAs that are regulated upon eIF5A depletion is *MYC* mRNA, where there is increased synthesis of an N-terminally extended proteoform. Importantly, the eIF5A-dependent pause site should be located close enough to the suboptimal start codon to promote its utilization upon eIF5A depletion, which is consistent with the queueing model. A similar role for eIF5A was also shown for the regulation of the AUU-initiated AZIN1 uORF [116]. The effect of eIF5A on non-AUG initiation was also documented in yeast and this type of regulation has been found during meiosis [121].

It is expected that any site-specific pause of elongating ribosomes may have similar effects on non-optimal start codons. Such ribosome pauses can be induced by rare codons, unavailability of cognate charged tRNAs or nascent chain-mediated ribosome slow down. Therefore, the amino acid sequence downstream of non-AUG or sub-optimal start codons may be a critical determinant of mRNA specific initiation (Fig. 3C).

## Conclusion

It is becoming increasingly clear that initiation at non-AUG codons is widely utilized in mammalian cells and significantly contributes to “hidden proteome” diversity. Such non-canonical start codons have a wide range of initiation efficiencies, from zero activity to efficiencies comparable with that of AUG codons. A specific nucleotide context is required for efficient non-AUG initiation which can be further enhanced by downstream stimulators (such as an RNA secondary structure), and, in some rare cases, such codons are recognized almost as well as AUG codons by the translation machinery, as the initiation factors that usually “sense” suboptimal codons fail to affect initiation on such “super-optimal” non-AUG starts [52]. Nevertheless, even the most efficiently initiated non-AUG codons appear to be “leaky” (Fig. 2, translation of downstream AUG initiated ORFs is evident).

Recent studies demonstrated that non-AUG initiation can be exceptionally sensitive to conditions that pause scanning ribosome progression. This includes downstream secondary structures which are likely difficult to unwind by conventional RNA helicase eIF4A activity, and slowly decoded triplets which may induce a queue of elongating 80S ribosomes. In this regard, non-AUG codons are more dependent on downstream sequences than AUG codons, where good nucleotide context is usually enough to provide efficient initiation. Downstream sequences can provide selective translation control of mRNA under various conditions. In our opinion, it is likely that specific non-AUG codons in 5′ leaders are among the most widespread and important genetically encoded regulatory elements in the mammalian genome. Identification of active non-AUG initiation start sites is essential for genome annotation and for the discovery of functional micropeptides and proteoforms [122].

The implication of non-AUG initiation in various human diseases, such as cancer [67, 68] and neurological disorders [69, 123], prompted many to suggest it as a therapeutic target. While affecting non-AUG initiation is unlikely to be specific due to the ubiquitous nature of this phenomenon, the sensitivity of particular non-AUG initiators to specific factors and conditions suggests that such an approach could hold some promise.

## Supplementary Information

**Additional file 1.** Peer review history.

## Data Availability

No supporting data or materials are provided for this manuscript.
