# Supplementary material for: Non-AUG translation initiation in mammals
Source: Genome Biol. 2022 May 9;23:111. doi: 10.1186/s13059-022-02674-2 (PMC9082881; doi:10.1186/s13059-022-02674-2)
Supplement: Supplementary file 1 — Additional file 1. Peer review history. [file 13059_2022_2674_MOESM1_ESM.docx]

**Review history**

**First round of review**

**Reviewer 1**

Andreev et al. provide a focused and detailed review on non-AUG translation in mammalian systems. The breath of cite literature and topics are appropriate, give credit to those in the field, and raises important questions that remain unanswered. The manuscript is well written and would be of wide interest, including to those in the translational control field and those whom are focused on the coding capacity of the mammalian genome. In total, the authors present a strong review of the field and do so in a non-biased manner.

The following critiques are more based on clarification and formatting:

1) The use of an apostrophe instead of a prime symbol is used not uniformly. Ideally, the prime sympol is more accurate.

2) A reference for the analysis stated in lines 56-58 should be added.

3) In regards to the referenced ribosome profiling datasets collected after harringtonine or lactimidomycin treatment, using "initiating ribosomes" may not be the most accurate. I understand the meaning behind it, but the drugs inhibit the first translocation step of elongation. They are often referred to as early elongation inhibitors. Using a different term than "initiating ribosomes" here would be more accurate for a wider audience.

4) The tRNA nomenclature is not uniform and is often not accurate. For example, Leu tRNAi is not conventional. In the references Starck et al. papers, is the Leu-tRNA used for initiation really a different tRNA than the Leu-tRNA used during elongation (like known for Met-tRNAi vs. Met-tRNAe).

5) In the "Nucleotide contexts that influence non-AUG initiation", I feel adding more details or examples to some statements would provide a better understanding for the audience. For example, line 194-195 and line 198-199.

6) In regards to cell stress and p-eEF2 (by eEF2K), most cell stress also inhibits initiation. Thus, PIC loading and queuing would be inhibited too. It may be worth pointing out this caveat.

7) The "Interplay between elongation and initiation rates can be important for non-AUG translation" section is very well written thoroughly covers the field. However, I feel the importance of Ivanov & Dever's paper (reference 101) should be presented more strongly and perhaps first. This work was published before the CHX story from Kearse et al. (2019) (reference 90).

8) For Figure 3C, should it be 43S instead of 48S? Isn't the 48S really just the 43S on the mRNA at the start codon? In this case, the mRNA already has an 80S on it, so it's not going to be observed as 48S.

9) The inserts in Figure 2 are a little blurry in this current file. This may not be an actual issue if the journal requires the actual figure file.

**Reviewer 2**

Here Andreev et al provides a thorough review of the subject- non-AUG translation in mammals. The topic covered is timely and the way they are arranged is logical. Recent works (some of them are not cited here, however) also highlight a common mechanistic basis between repeat-associated non-AUG translation and more general non-AUG translation as discussed here, so a review like this would be appreciated in the field. However, I have several concerns related to accuracy, balance and relevance. My major concern is a weak reference to founding works in prokaryotes and yeasts. Though this review covers mammals, non-AUG translation (GUG and UUG initiation) is common in prokaryotes (bacteria and archaea). Some of the historical works instrumental to current understanding of eukaryotic translation should be referenced to help introduce readers to this subject. My recommendation about this is discussed below in the major concern. My other recommendations including better reference to founding yeast works are discussed further below related to Pages 10 and 11 and elsewhere.

Major concern: Suggested paragraphs are described below, to be inserted in place of the last sentence of the second paragraph of page 2.

"Most likely for this reason, the stringent control of the base-pairing in the P-site is compromised. Prokaryotic ribosomes (both from Bacteria and Archaea) locate translation start sites through a Shine-Dalgarno sequence preceding the start codon and initiate translation from GUG and UUG codons besides AUG; GUG and UUG codons can base-pair with fMet-tRNAifMet anticodon, allowing wobble base-pairing at the first position in the P-site (new Ref #1). Some riboswitches take advantage of the ability to completely inhibit translation from a weak GUG start codon through a small stem-loop structure (new ref #2)."

In contrast, eukaryotes evolved a complex initiation pathway, allowing stringent initiation from the AUG start codon. During the scanning process, initiation from codons other than AUG is strictly prohibited [4], in part through inhibiting wobble base-pairing at the first position (new ref #3). (In addition to the identity….)"

New references:
1. https://www.tandfonline.com/doi/full/10.4161/trla.28387
2. https://www.sciencedirect.com/science/article/pii/S0021925818542642
3. https://journals.plos.org/ploscompbiol/article?id=10.1371/journal.pcbi.1009068

Other concerns with recommended additional citations:

Abstract

"This is enabled by the leaky scanning mode of translation initiation" - here," this" refers to non-canonical initiation. However, this is a misleading statement, because non-canonical initiation is enabled by mis-initiation by canonical initiation and not the leaky scanning. Instead, leaky scanning of non-canonical initiation allows the main CDS to be translated at a high rate. Related to this, misuse of "leaky translation" is found throughout and must be corrected.

Page 2

"Presumably, the lack of stringent control of the base-pairing in the P-site enables Met-tRNAi to recognize codons other than AUG as starts, albeit less efficiently."

The phrase "the lack of stringent control" is problematic and underestimates what more is learned about the stringent mechanism. The eukaryotic decoding system has evolved to confer a stringent initiation, much more stringent than prokaryotic ones which allow GUG and UUG initiation (This comparison is well described in this review).
https://www.tandfonline.com/doi/full/10.4161/trla.28387

However, it is the evolution of cis-regulatory elements that confers a wide range of efficiencies of initiation from various codons under various contexts. The codon-specific differences with respect to control by eIF1 has been noted in the following recent work utilizing an advanced molecular dynamics simulation study.
https://journals.plos.org/ploscompbiol/article?id=10.1371/journal.pcbi.1009068

Page 3

"due to the minimal topological constrains of its codon-anticodon duplex"
The aforementioned work by Kameda et al is in favor of this statement. Citation of this work at the end of this paragraph is appropriate here.

"early studies based on reporter constructs indicated that nucleotides at +5 and +6 are important for the efficiency of non-AUG initiation [69, 70]."
Add the reference [14] here for the importance of +5 and +6 positions as examined by a reporter construct and mention "a downstream stem-loop structure" citing Kozak work [79]. This is mentioned later in the manuscript, but it is appropriate to introduce it here.

"However, it is believed that most translated uORFs either serve regulatory purposes or are mostly inconsequential tolerances having on average only a mild inhibitory effect on downstream translation [22]. The average effect is mild because most uAUGs are in a weak context and are therefore leaky and also because ribosomes terminating after translation of short ORFs are often capable of reinitiating [23].": This segment describing uORF is inaccurate and misleading. It fails to dissect two different concepts - weak initiation allowing leaky scanning and re-initiation that happens after translation termination of an uORF. My suggested revision based on a standardized, traditional understanding is as follows:

"However, most translated uORFs either serve regulatory purposes or are not inhibitory to downstream translation due to their weak translation initiation [22]. In the regulatory uORFs, ribosomes terminating after translation of a uORF are capable of "re-initiation" as they are anchored to the same mRNA and resume scanning, rather than being recycled for translation of other mRNAs [23]. The best studied example of regulatory uORFs is found in translational control of yeast GCN4 involving four uORFs, of which the first initiated strongly allows re-initiation of downstream ORFs; the choice of re-initiation sites is determined by eIF2 phosphorylation, limiting the availability of active eIF2/GTP/Met-tRNAiMet terminay complex [23]. Similar regulatory uORFs are found in mammalian ATF4 and many other genes throughout eukaryotes (new references below)."
https://link.springer.com/article/10.1007/s00294-020-01149-w
https://academic.oup.com/nar/article/48/16/8977/5876285?login=true

Page 4

"Hann et al [28] detected two c-Myc proteoforms that originate from a single mRNA with a single AUG-initiated ORF. It was subsequently shown that N-terminally extended Myc (p67, myc1) starts from a CUG codon located 15 triplets upstream of the main ORF AUG."

It should be noted that this earlier work addressed functional difference between the AUG-vs CUG-initiated isoforms merely by an in vitro transcription assay with a very limited set of template samples. The genome-wide effect of these isoforms was just recently verified in the following work that shows the former being likely more favorable for a phenotype supportive of cancer progression than the latter.
https://www.sciencedirect.com/science/article/pii/S2352396419303664

Page 7

"Long repetitive sequences have been shown to stimulate translation initiation upstream, more details on the molecular processes and associated disease etiology can be found in several reviews dedicated to this topic [65-67]."

The following recent works make RAN translation mechanistically more akin to general non-AUG translation. Therefore, this topic is more relevant in this review and needs to be expanded. Also, this sentence is not accurate and must be corrected (Is the repeat shown to stimulate translation initiation? Of what translation?).

"Long repetitive sequences have been shown to be translated by initiation from a repeat codon within the repeat or a near-cognate start codon upstream of the repeats. While details on the molecular processes and associated disease pathology can be found in several reviews [65-67], recent works highlight that RAN translation is mechanistically more similar to general non-AUG translation, involving common 5'cap dependence and regulation as discussed next."
https://www.sciencedirect.com/science/article/pii/S2211124721007749
https://www.sciencedirect.com/science/article/pii/S0896627313002730
https://www.sciencedirect.com/science/article/pii/S1097276516001787

Page 8

"The development of a high throughput approach for massively parallel variant analysis of initiation efficiency, FACS-seq, allowed estimation of all individual context variants in cells."

As this is mentioned later, the following must be stated here related to this work:

"In this screening, -4 to -1 and +4 positions around the start codon of a GFP reporter was randomized, and its expression from individual constructs was measured all by FACS. Subsequently, the reporter DNA molecules were amplified and sequenced"

Page 10
"when three genes, sui1, sui2, and SUI3 (suppressors of initiator codon mutants) was discovered, which when mutated confer the ability to translate mutant HIS4 despite the absence of an AUG start codon [71, 72]. These genes appear to encode initiation factors eIF1 and two out of the three subunits of eIF2, respectively."
This statement is misleading (these genes appear to..? no, definitely they do) and inaccurate, and must be corrected as follows:
"when the Donahue group performed a genetic screening for sui/SUI alleles (suppressors of initiator codon mutants), taking advantage of mutants' ability to confer translation of a mutant his4 allele despite the absence of an AUG start codon [71, 72]. These genes encode initiation factors eIF1, eIF5 and all three subunits of eIF2."
SUI4 and SUI5 encode eIF2gamma and eIF5, respectively. The last sentence must cite this following work.
http://genesdev.cshlp.org/content/11/18/2396.long

"The rate of dissociation of eIF1 from 48S seems to be a crucial determinant of start codon recognition [75]."
Following this, a sentence like this nicely connects to the role of eIF5 as well as another important factor, eIF3, that is very important but was not mentioned in the review.

"Subsequently, it was shown that accurate translation initiation entails control of binding and release of eIF1 by other factors such as eIF3 and eIF5."
Cite the following work on eIF3(c) and eIF5.
https://www.sciencedirect.com/science/article/pii/S2211124717302504
https://journals.asm.org/doi/full/10.1128/MCB.24.21.9437-9455.2004
https://www.pnas.org/content/102/45/16164.short

Page 11

"Both proteins are homologous to eIF5 at its N-terminal domain but lack its C terminal GTPase activating GAP domain."
Correct this wrong statement as follows:
"Both proteins are homologous to eIF5 at its C-terminal domain but lack its N terminal GTPase activating GAP domain."
Cite the following works related to genetic/two-hybrid dissection of eIF5 structures.
https://journals.asm.org/doi/full/10.1128/MCB.25.13.5480-5491.2005
http://genesdev.cshlp.org/content/14/19/2534.short
https://www.sciencedirect.com/science/article/pii/S2211124712001209


"Indeed, upon eIF1 deprivation, only 245 out of 10,000 actively translated transcripts showed changes in translation."

There is another good reason for this, that are not mentioned in this manuscript: There are two copies of eIF1 in human genome, EIF1 and EIF1B. Here is a serious mis-nomenclature making it not like eIF1 (next to eIF4G2 being DAP5/p97/NAT1), but EIF1B is expressed well and is very similar to and functionally identical to EIF1. This point must be described earlier in this chapter.

Fig. 1

Abbreviation PANTS is not necessary and should be eliminated from the figure and the text. N-terminal extension is sufficient unless there is a specific functional definition of a certain kind is needed.

"RAN-associated non-AUG initiation" is an incorrect word, as RAN stands for "repeat-associated non-AUG". Rephrase as "repeat-associated non-AUG (ATG) initiation" or "- translation".

**Authors’ response to reviewers**

Reviewer reports:

Reviewer #1: Andreev et al. provide a focused and detailed review on non-AUG translation in mammalian systems. The breath of cite literature and topics are appropriate, give credit to those in the field, and raises important questions that remain unanswered. The manuscript is well written and would be of wide interest, including to those in the translational control field and those whom are focused on the coding capacity of the mammalian genome. In total, the authors present a strong review of the field and do so in a non-biased manner.

Response: We thank the reviewer for high evaluation of our work.

The following critiques are more based on clarification and formatting:

1) The use of an apostrophe instead of a prime symbol is used not uniformly. Ideally, the prime sympol is more accurate.

Response: Thank you for spotting this. This change has now been incorporated throughout.

2) A reference for the analysis stated in lines 56-58 should be added.

Response: We searched for AUG codons in the latest RefSeq and Gencode releases by ourselves. To make it clear, we added “we searched current transcript annotations and found that”).

2) In regards to the referenced ribosome profiling datasets collected after harringtonine or lactimidomycin treatment, using "initiating ribosomes" may not be the most accurate. I understand the meaning behind it, but the drugs inhibit the first translocation step of elongation. They are often referred to as early elongation inhibitors. Using a different term than "initiating ribosomes" here would be more accurate for a wider audience.

Response: We agree with the reviewer and now substituted “initiating ribosomes” with “ribosomes enriched at start codons”

3) The tRNA nomenclature is not uniform and is often not accurate. For example, Leu tRNAi is not conventional. In the references Starck et al. papers, is the Leu-tRNA used for initiation really a different tRNA than the Leu-tRNA used during elongation (like known for Met-tRNAi vs. Met-tRNAe).

Response: We thank the reviewer for spotting this and we now corrected and unified tRNA nomenclature.

5) In the "Nucleotide contexts that influence non-AUG initiation", I feel adding more details or examples to some statements would provide a better understanding for the audience. For example, line 194-195 and line 198-199.

Response: We have now added a sentence which describes the positive effect of A and U at the +5 and +6 position for efficient initiation at non-AUG codons

6) In regards to cell stress and p-eEF2 (by eEF2K), most cell stress also inhibits initiation. Thus, PIC loading and queuing would be inhibited too. It may be worth pointing out this caveat.

Response: Thank you for pointing out this caveat, we now added the following sentence: “However, as stress conditions may also result in decreased initiation, this can counteract ribosome queue formation caused by slow elongation.”

7) The "Interplay between elongation and initiation rates can be important for non-AUG translation" section is very well written thoroughly covers the field. However, I feel the importance of Ivanov & Dever's paper (reference 101) should be presented more strongly and perhaps first. This work was published before the CHX story from Kearse et al. (2019) (reference 90).

Response: We agree that the Ivanov & Dever study is very important and provided major insight into our understanding that elongation rate can impact initiation. However, we think that placing the Ivanov & Dever reference before Kearse et al would compromise the logic flow of this section. To solve the issue, we extended the sentence that cites Ivanov & Dever with the following:

“… thus confirming Dinesh-Kumar et al {Dinesh-Kumar et al 1993 reference} hypothesis and providing mechanistic explanation for the later observation of global non-AUG initiation increase in response to elongation inhibitors {Kearse et al 2019 reference}”

8) For Figure 3C, should it be 43S instead of 48S? Isn't the 48S really just the 43S on the mRNA at the start codon? In this case, the mRNA already has an 80S on it, so it's not going to be observed as 48S.

Response: We agree that the complex with 80S on mRNA would not migrate as 48S. However, the complex of 43S on the mRNA at the start codon is often referred to as 48S and thus we think that it will be more appropriate to refer to it as to 48S. Consider two 80S ribosomes on mRNA, they would migrate as a disome, but it is still clearer to refer to each of them as 80S.

9) The inserts in Figure 2 are a little blurry in this current file. This may not be an actual issue if the journal requires the actual figure file.

Response: Thank you for pointing this out, we will try to improve the quality of this figure if the manuscript is accepted.

Reviewer #2: Here Andreev et al provides a thorough review of the subject- non-AUG translation in mammals. The topic covered is timely and the way they are arranged is logical. Recent works (some of them are not cited here, however) also highlight a common mechanistic basis between repeat-associated non-AUG translation and more general non-AUG translation as discussed here, so a review like this would be appreciated in the field.

Response: We thank the reviewer for high evaluation of our work and for a large number of suggestions and corrections, some of which we find very useful.

However, I have several concerns related to accuracy, balance and relevance. My major concern is a weak reference to founding works in prokaryotes and yeasts. Though this review covers mammals, non-AUG translation (GUG and UUG initiation) is common in prokaryotes (bacteria and archaea). Some of the historical works instrumental to current understanding of eukaryotic translation should be referenced to help introduce readers to this subject. My recommendation about this is discussed below in the major concern. My other recommendations including

better reference to founding yeast works are discussed further below related to Pages 10 and 11 and elsewhere.

Response: We agree that our focus on non-AUG initiation in mammals may create an impression that it occurs only in mammals. To avoid such misinterpretation, we added the following two sentences at the end of our introduction:

“While non-AUG initiation is known to take place in prokaryotes, as well as in lower eukaryotes such as yeast, this is outside of the scope of this review. We mention relevant studies in yeast in a historical context only when they provide important mechanistic insights.”

Major concern: Suggested paragraphs are described below, to be inserted in place of the last sentence of the second paragraph of page 2.

"Most likely for this reason, the stringent control of the base-pairing in the P-site is compromised. Prokaryotic ribosomes (both from Bacteria and Archaea) locate translation start sites through a Shine-Dalgarno sequence preceding the start codon and initiate translation from GUG and UUG codons besides AUG; GUG and UUG codons can base-pair with fMet-tRNAifMet anticodon, allowing wobble base-pairing at the first position in the P-site (new Ref #1). Some riboswitches take advantage of the ability to completely inhibit translation from a weak GUG start codon through a small stem-loop structure (new ref #2)."

In contrast, eukaryotes evolved a complex initiation pathway, allowing stringent initiation from the AUG start codon. During the scanning process, initiation from codons other than AUG is strictly prohibited [4], in part through inhibiting wobble base-pairing at the first position (new ref #3). (In addition to the identity….)"

New references:

1. <https://www.tandfonline.com/doi/full/10.4161/trla.28387>

2. <https://www.sciencedirect.com/science/article/pii/S0021925818542642>

3. <https://journals.plos.org/ploscompbiol/article?id=10.1371/journal.pcbi.1009068>

Response: We thank the Reviewer for this suggestion and respect the Reviewer’s opinion, however we decided not to follow this suggestion for several reasons.

First, the mechanistic aspect of prokaryotic initiation is indeed very different from eukaryotic initiation. The comprehensive coverage of the initiation in bacteria in this review would not be possible without its substantial expansion, and this ultimately will destroy its focus. Translation initiation and start codon selection fidelity in bacteria is a separate and a very interesting topic which deserve to be covered in a dedicated publication, especially in the light of a recent study which shed new light in the role of Shine Dalgarno sequence in the selection of start codons in bacteria(Saito et al 2020 eLife DOI: 10.7554/eLife.55002). Of note, many bacterial species lost anti-Shine-Dalgarno sequences (Nikolaeva et al Mol Biol Evol DOI: 10.1093/molbev/msaa184.)

Next, the reviewer suggests that initiation in eukaryotes is more stringent than in prokaryotes, but we think that we can not make such conclusion. On one hand, in contrast to eukaryotes, there is significant proportion of annotated protein coding genes in bacteria that initiate at non-AUG codons. On the other hand, genome wide approaches identify many more non-AUG starts in mammals (Lee et al PNAS 2012 DOI: 10.1073/pnas.1207846109; Ingolia et al 2011 Cell DOI: 10.1016/j.cell.2011.10.002) and yeast (Spealman et al 2018 Genome Res DOI: 10.1101/gr.221507.117,) than in bacteria (Nakahigashi et al 2016 DNA Res DOI: 10.1093/dnares/dsw008; Meydan et al 2020). We believe it is not accurate to compare the fidelity of non-AUG initiation in prokaryotes and eukaryotes. The difference in the number of annotated non-AUG codons in bacterial and eukaryotic genomes is largely due to the differences in annotation pipelines and may not be biological. We are involved in collaboration with Gencode which aims to improve annotation of start codons in the human genome using available data. This will substantially increase the number of non-AUG initiated proteoforms in the human genome. This is one of the reasons for writing this review.

Other concerns with recommended additional citations:

Abstract

"This is enabled by the leaky scanning mode of translation initiation" - here," this" refers to non-canonical initiation. However, this is a misleading statement, because non-canonical initiation is enabled by mis-initiation by canonical initiation and not the leaky scanning. Instead, leaky scanning of non-canonical initiation allows the main CDS to be translated at a high rate. Related to this, misuse of "leaky translation" is found throughout and must be corrected.

Response: The reviewer misinterpreted our text, “this” refers to “non-canonical translation” from the previous sentence not “non-canonical initiation”.

We haven’t used a term “leaky translation” in our review, only “leaky scanning” which describes the process when ribosomes that failed to initiate at a start codon (AUG or non-AUG) continue scanning. Since non-AUG are generally recognized less efficiently than AUG, they are leakier. Since the topic of our review is on initiation at non-AUG codons, the leaky scanning mode of translation initiation is crucial and indeed is often mentioned.

However, in principle, the multiplicity of start codons is also enabled by re-initiation, even though it is less relevant to non-AUG initiation in comparison with leaky scanning. To take it into account we added a word “partly” to the abstract (the 100 words limit does not allow us to provide detail explanation of re-initiation, but we also think it is not needed).

Page 2

"Presumably, the lack of stringent control of the base-pairing in the P-site enables Met-tRNAi to recognize  codons other than AUG as starts, albeit less efficiently."

The phrase "the lack of stringent control" is problematic and underestimates what more is learned about the stringent mechanism. The eukaryotic decoding system has evolved to confer a stringent initiation, much more stringent than prokaryotic ones which allow GUG and UUG initiation (This comparison is well described in this review).

<https://www.tandfonline.com/doi/full/10.4161/trla.28387>

However, it is the evolution of cis-regulatory elements that confers a wide range of efficiencies of initiation from various codons under various contexts. The codon-specific differences with respect to control by eIF1 has been noted in the following recent work utilizing an advanced molecular dynamics simulation study.

<https://journals.plos.org/ploscompbiol/article?id=10.1371/journal.pcbi.1009068>

Response: When we referred to “the lack of stringent control” we referred to the control of base-pairing between codon and anticodon in the P-site by the ribosome itself that is less stringent than that in the A-site. We believe that the Reviewer misunderstanding is caused by frequent use of the word stringency in the literature in its relation to the process of start codon selection. To avoid misinterpretation we changed “the lack of stringent control of the base-pairing ” to “reduced control of the base-pairing geometry”. The comparison of prokaryotes with eukaryotes is irrelevant here because we compare the decoding in the P-site to the decoding in the A-site.

Page 3

"due to the minimal topological constrains of its codon-anticodon duplex"

The aforementioned work by Kameda et al is in favor of this statement. Citation of this work at the end of this paragraph is appropriate here.

Response: The work by Kameda et al is indeed relevant to the question why CUG is the most efficient non-AUG codon in mammals and we mention it now as Reviewer suggested.

"early studies based on reporter constructs indicated that nucleotides at +5 and +6 are important for the efficiency of non-AUG initiation [69, 70]."

Add the reference [14] here for the importance of +5 and +6 positions as examined by a reporter construct and mention "a downstream stem-loop structure" citing Kozak work [79]. This is mentioned later in the manuscript, but it is appropriate to introduce it here.

Response: We believe that adding reference 14 is not really relevant here, as we describe non-AUG initiation and 14 is about AUG initiation. The reason of this sentence is to highlight that it was recognized a long time ago that nucleotides downstream of non-AUG at +5 and +6 have stronger impact on translation than in case of AUG codons.

"However, it is believed that most translated uORFs either serve regulatory purposes or are mostly inconsequential tolerances having on average only a mild inhibitory effect on downstream translation [22]. The average effect is mild because most uAUGs are in a weak context and are therefore leaky and also because ribosomes terminating after translation of short ORFs are often capable of reinitiating [23].": This segment describing uORF is inaccurate and misleading. It fails to dissect two different concepts - weak initiation allowing leaky scanning and re-initiation that happens after translation termination of an uORF. My suggested revision based on a standardized, traditional understanding is as follows:

"However, most translated uORFs either serve regulatory purposes or are not inhibitory to downstream translation due to their weak translation initiation [22]. In the regulatory uORFs, ribosomes terminating after translation of a uORF are capable of "re-initiation" as they are anchored to the same mRNA and resume scanning, rather than being recycled for translation of other mRNAs [23]. The best studied example of regulatory uORFs is found in translational control of yeast GCN4 involving four uORFs, of which the first initiated strongly allows re-initiation of downstream ORFs; the choice of re-initiation sites is determined by eIF2 phosphorylation, limiting the availability of active eIF2/GTP/Met-tRNAiMet terminay complex [23]. Similar regulatory uORFs are found in mammalian ATF4 and many other genes throughout eukaryotes (new references below)."

<https://link.springer.com/article/10.1007/s00294-020-01149-w>

<https://academic.oup.com/nar/article/48/16/8977/5876285?login=true>

Response: We agree with the reviewer that we often cannot dissect the reason of translation downstream of an uORF. The ribosomes can reach downstream locations either due to leaky scanning or due to reinitiation, or combination of both. We feel that the comment relates to a possibility to interpret our text as if we claim that there is a relationship between “leaky scanning and reinitiation”. We do not make such a claim and in order to avoid misinterpretation we rephrased the last sentences as the following:

“However, it is believed that most translated uORFs only have a mild inhibitory effect on downstream translation {Chew, 2016 #266}. The average inhibitory effect of uORFs is mild either because most uORF starts are leaky (non-AUG or AUG in a weak context), or also because ribosomes terminating after translation of short ORFs are often capable of reinitiating {Gunisova, 2018 #287}.

We agree with the reviewer that GCN4 is one of the best studied examples of regulatory uORFs in yeast. However, there is a very large number of well-studied and highly versatile regulatory mechanisms enabled by uORFs in humans and we think it is more important to mention them rather than get into the details of a specific mechanism studied in yeast. Moreover, describing the regulation of GCN4 will require us to comprehensively describe the role of eIF2 phosphorylation in translation control, which is not really relevant to non-AUG initiation.

Page 4

"Hann et al [28] detected two c-Myc proteoforms that originate from a single mRNA with a single AUG-initiated ORF. It was subsequently shown that N-terminally extended Myc (p67, myc1) starts from a CUG codon located 15 triplets upstream of the main ORF AUG."

It should be noted that this earlier work addressed functional difference between the AUG-vs CUG-initiated isoforms merely by an in vitro transcription assay with a very limited set of template samples. The genome-wide effect of these isoforms was just recently verified in the following work that shows the former being likely more favorable for a phenotype supportive of cancer progression than the latter.

<https://www.sciencedirect.com/science/article/pii/S2352396419303664>

Response: We thank the reviewer for mentioning this study and we now include this reference in the modified sentence.

Page 7

"Long repetitive sequences have been shown to stimulate translation initiation upstream, more details on the molecular processes and associated disease etiology can be found in several reviews dedicated to this topic [65-67]."

The following recent works make RAN translation mechanistically more akin to general non-AUG translation. Therefore, this topic is more relevant in this review and needs to be expanded. Also, this sentence is not accurate and must be corrected  (Is the repeat shown to stimulate translation initiation? Of what translation?).

"Long repetitive sequences have been shown to be translated by initiation from a repeat codon within the repeat or a near-cognate start codon upstream of the repeats. While details on the molecular processes and associated disease pathology can be found in several reviews [65-67], recent works highlight that RAN translation is mechanistically more similar to general non-AUG translation, involving common 5'cap dependence and regulation as discussed next."

<https://www.sciencedirect.com/science/article/pii/S2211124721007749>

<https://www.sciencedirect.com/science/article/pii/S0896627313002730>

<https://www.sciencedirect.com/science/article/pii/S1097276516001787>

Response: We agree with the reviewer’s suggestion that it is important to mention that RAN translation is mediated through scanning and is regulated by canonical translation factors and regulators, so we now include the following sentence with three new references to mentioned studies.

“However, it is important to note that during RAN translation recognition of non-AUG codons seems to occur via the standard cap-dependent mechanism and involves canonical initiation factors and regulators [72][73][74].

Page 8

"The development of a high throughput approach for massively parallel variant analysis of initiation efficiency, FACS-seq, allowed estimation of all individual context variants in cells."

As this is mentioned later, the following must be stated here related to this work:

"In this screening, -4 to -1 and +4 positions around the start codon of a GFP reporter was randomized, and its expression from individual constructs was measured all by FACS. Subsequently, the reporter DNA molecules were amplified and sequenced"

Response: We agree with the reviewer that the FACS technique requires clarification, so now we include more detail on the methodology. We chose not to specifically mention the 4 to -1 and +4 positions since we refer to two separate FACS seq studies, in case of AUG the authors randomized more positions than in case of non-AUG codons.

Page 10

"when three genes, sui1, sui2, and SUI3 (suppressors of initiator codon mutants) was discovered, which when mutated confer the ability to translate mutant HIS4 despite the absence of an AUG start codon [71, 72]. These genes appear to encode initiation factors eIF1 and two out of the three subunits of eIF2, respectively."

This statement is misleading (these genes appear to..? no, definitely they do) and inaccurate, and must be corrected as follows:

"when the Donahue group performed a genetic screening for sui/SUI alleles (suppressors of initiator codon mutants), taking advantage of mutants' ability to confer translation of a mutant his4 allele despite the absence of an AUG start codon [71, 72]. These genes encode initiation factors eIF1, eIF5 and all three subunits of eIF2."

SUI4 and SUI5 encode eIF2gamma and eIF5, respectively. The last sentence must cite this following work.

<http://genesdev.cshlp.org/content/11/18/2396.long>

Response: We thank the reviewer for spotting this inaccurate sentence, it is now corrected and additional reference is included.

"The rate of dissociation of eIF1 from 48S seems to be a crucial determinant of start codon recognition [75]."

Following this, a sentence like this nicely connects to the role of eIF5 as well as another important factor, eIF3, that is very important but was not mentioned in the review.

"Subsequently, it was shown that accurate translation initiation entails control of binding and release of eIF1 by other factors such as eIF3 and eIF5."

Cite the following work on eIF3(c) and eIF5.

<https://www.sciencedirect.com/science/article/pii/S2211124717302504>

<https://journals.asm.org/doi/full/10.1128/MCB.24.21.9437-9455.2004>

<https://www.pnas.org/content/102/45/16164.short>

Response: This is very good suggestion; we now include this sentence and the references in the text as per Reviewer suggestion.

Page 11

"Both proteins are homologous to eIF5 at its N-terminal domain but lack its C terminal GTPase activating GAP domain."

Correct this wrong statement as follows:

"Both proteins are homologous to eIF5 at its C-terminal domain but lack its N terminal GTPase activating GAP domain."

Cite the following works related to genetic/two-hybrid dissection of eIF5 structures.

<https://journals.asm.org/doi/full/10.1128/MCB.25.13.5480-5491.2005>

<http://genesdev.cshlp.org/content/14/19/2534.short>

<https://www.sciencedirect.com/science/article/pii/S2211124712001209>

Response: We thank the reviewer for spotting this mistake, it is now corrected as suggested. The corresponding references are now added.

"Indeed, upon eIF1 deprivation, only 245 out of 10,000 actively translated transcripts showed changes in translation."

There is another good reason for this, that are not mentioned in this manuscript: There are two copies of eIF1 in human genome, EIF1 and EIF1B. Here is a serious mis-nomenclature making it not like eIF1 (next to eIF4G2 being DAP5/p97/NAT1), but EIF1B is expressed well and is very similar to and functionally identical to EIF1. This point must be described earlier in this chapter.

Response: This is very interesting point. We are aware about existence of eIF1B, moreover in Fijałkowska et al the authors investigated the role of eIF1B in translation. While it looks likely that eIF1B can substitute for eIF1, it is currently not clear to us whether eIF1B is completely equivalent to eIF1. After all, they are similar but not identical (92% identity). In addition, eIF1KO and eIF1B KO seem to induce different changes in translatome (Fijałkowska et al, Fig 3C). Finally, and most puzzlingly for us, these authors mentioned that they did not find any apparent effect of eIF1B and eIF1 double knockdown on cell proliferation. Anyway, we agree that eIF1B should be mentioned and now include it in the new version of the manuscript as the following:

Notably, eIF1B, the paralog of eIF1 which shares 92% sequence identity, may also be implicated in start codon selection and the regulation of start codon selection stringency {Fijalkowska, 2017 #140}

Fig. 1

Abbreviation PANTS is not necessary and should be eliminated from the figure and the text. N-terminal extension is sufficient unless there is a specific functional definition of a certain kind is needed.

Response: Whether something is an extension or truncation is relative. Consider a proteoform with two start codons: Upstream Start (Ustart) and Downstream Start (Dstart). If Dstart is annotated and Ustart is newly discovered we would refer to such case as an extension. However, if it is the other way around, we will have to refer to it as a truncation. But the difference lies in the annotation of these proteoforms, not in its biological properties. Indeed, many proteoforms truncated relative to the annotation have been discovered including where the truncated proteoform initiates with non-AUG in mRPL18.

Thus, we believe that the term N-terminal extension is limited and misleading because it depends on historical annotations, there are even situations when one annotation catalog annotates Ustart as a start while the other annotates Dstart as a start. We came across such situation in our own recent study (Benitez-Cantos et al 2020 doi:10.1101/gr.257352.119).

Therefore, to avoid the confusion caused by the use of terms such as truncated or extend proteoforms we introduced a new term PANT for Proteoforms with Alternative N Termini. This term is annotation independent and is objective. We believe that the term PANT will be adopted by the scientific community for the reasons described above.

"RAN-associated non-AUG initiation" is an incorrect word, as RAN stands for "repeat-associated non-AUG". Rephrase as "repeat-associated non-AUG (ATG) initiation" or "- translation".

Response: Thank you for spotting this inaccurate description, it is now corrected.

**Second round of review**

**Reviewer 1**

The authors have made the manuscript stronger. I have no other concerns.

**Reviewer 2**

I appreciate very careful and through responses by the authors. I agree with most of the points, but I still have a trouble. I hope this message goes through to the authors.

Abstract: I believe the authors' use of the term "leaky scanning" is incorrect. I tried to find the best definition but the typical one that agrees with my understanding is as follows:

"Leaky scanning is a phenomenon in which a weak initiation codon triplet on mRNA is sometimes skipped by ribosome in translation initiation. The 40S ribosomal subunit continues scanning to further initiation codon. The weak initiation codon can be an ACG, or an ATG in a weak Kozak consensus context.
This way a mRNA can encode for several different proteins if the AUG are not in frame, or for proteins with different N-terminus if the AUG are in the same frame."

https://viralzone.expasy.org/1976#:~:text=Leaky%20scanning%20is%20a%20phenomenon,a%20weak%20Kozak%20consensus%20context.

In contrast, the present abstract states:

"This so-called noncanonical translation is largely due to the plurality of start sites within the same RNA. This is partly enabled by the leaky scanning mode of translation initiation, where some scanning ribosomes failing to recognize potential start codons allow downstream initiation. Here we discuss our current understanding of translation initiation at non-AUG codons which are intrinsically leaky due to their inefficiency."

Specifically, the statement "This (non-AUG translation) is partly enabled by the leaky scanning mode" is incorrect. If you rephrase this, it is clear. "Leaky scanning, or skipping of weak start codons, enables non-AUG translation" - this does not make sense! In Introduction, the authors correctly state that "the reduced control of the base-pairing geometry in the P-site enables Met-tRNAi to recognize (weak start codons)". In sum, the ribosomes (or its intrinsic inaccuracy or imperfect "strictness" in the authors term), not leaky scanning, enables translation of weak start codons. I agree that non-AUG translation enables leaky scanning, but not vice versa.

My best advice on the abstract is to avoid using the term "leaky scanning" in the earlier sentences to define non-AUG translation. Use this term as the means to allow a mRNA to code for multiple proteins (as correctly described in the aforementioned website). I checked through and found that other parts of the manuscript use the term leaky scanning or leaky correctly.

Second, we probably don't agree on the mechanism leading to fundamental differences and similarities on start codon selection by bacterial and eukaryotic ribosomes. I agree that this has to be handled elsewhere in a review. When you state "While non-AUG initiation is known to take place in prokaryotes, as well as in lower eukaryotes such as yeast and in plants, this is outside the scope of this review", however, I recommend to cite relevant reviews, such as (Hinnebusch, Dever et al. 2007, Asano 2014). This helps to give a wider perspective to the readers.

Suggested citations:

Asano, K. (2014). "Why is start codon selection so precise in eukaryotes?" Translation 2: e28387.

Hinnebusch, A. G., et al. (2007). Mechanism of translation initiation in the yeast Saccharomyces cerevisiae. Translational Control in Biology and Medicine. M. B. Mathews, N. Sonenberg and J. W. B. Hershey. Cold Spring Harbor, NY, Cold Spring Harbor Lab Press: 225-268.

**Authors’ response to reviewers**

We appreciate the comments of the second Reviewer, and we added the references suggested. We are particularly grateful to the second Reviewer for the explanation of the Reviewer's objection to the use of leaky scanning in the abstract as we now understand the source of the misunderstanding. We are in full agreement with the Reviewer regarding the definition of leaky scanning. However, it appears that the Reviewer believes that we invoke leaky scanning as an explanation of non-AUG initiation, which, of course, is not the case. The leaky scanning is mentioned to explain the existence of multiple starts, including multiple AUG starts. While we thought that our abstract was clear, this confusion indicates that it was not. Therefore, we altered the abstract in the way that should make it clear, that multiple starts exist as a result of leaky scanning and the relevance is because non-AUG starts are particularly leaky.
